# The Fracture of Platinum Pins in Teeth and the Cracking of Teeth in Soldering

**Published:** 1898-03

**Authors:** E. Duval


ARTICLE VII.

### THE FRACTURE OF PLATINUM PINS IN TEETH AND THE CRACKING OF TEETH IN SOLDERING.

BY E. DUVAL.

The fracture of platinum pins in teeth and the cracking of teeth in soldering is a difficulty, or rather annoyance, which occurs at some time or other to all interested in the mechanical aspect of dentistry. The following views, therefore, on this question, of some twenty men experts at the bench and of long experience may be of interest:

B.—Will solder 100 out of 100 teeth without accident. He generally bends pins towards root, sometimes rivets and sometimes bends only. Thinks some men are scanty with their backing, which should cover the whole tooth.

G.—Always rivets the teeth, but only slightly, and is very careful to get the heat up very gradually. Countersinks side of rivet. If a flat tooth rides on a stump, the chances are that the teeth will give in a short time.

P.—Has some teeth break, but does not think it is always the fault of the tooth, but considers that more break now than used to. Always rivets teeth, and countersinks side of rivet. There is great danger if teeth are touching on plate, they should never touch. Punches holes on boxwood, which causes depression. Depressed side goes next to tooth. The bur on the other side is filed away, and the hole countersunk.

L.—Always rivets, except where backing goes against vulcanite; pins are then left intact, only they are bent so as to form a hold in the vulcanite. Sometimes teeth crack across pins.

P. G.—Punches holes; and does not rivet; only slightly bends the pins together, and does not countersink. Has met with fractures of pins, and thinks them due to peculiarity of bit .

J.—Never has any teeth come off; does not rivet before soldering. Solders on plaster and sand mixed.

W. J.—Bends pins after punching holes, and lightly taps down with riveting hammer; then runs file over pins. He fits his tooth after backing is on, not before. Invests in plaster and sand. Carefully heats up. Rarely or never has accidents; might have three in a year among some thousands used.

S.—Used to rivet teeth, but owing to fractures and accidents had discontinued the practice. Prefers backing, countersinks holes both sides, and then without cutting off pins "nicks" them the way he bends them close to the backing, so that they come to lie at perfect right angles over the backing. Uses large numbers of teeth, and practically never has an accident.

J. P.—Does not find one tooth in a thousand break, nor the pins. Punches holes in backing, does not countersink, nor rivet, nor cut pins, but simply bends them rootwards to hold backing firm. Greatest danger is in the heating. Makes his cases nearly red-hot.

C.—Always cuts his pins short, and then splits them across, and bends them longitudinally. Countersinks slightly on both sides. Thinks accidents happen through over-heating, as on cracked or broken teeth solder can be seen on the side of backing touching tooth. Very rarely meets with fractures—practically never.

B. W.—Rarely cuts pins of teeth to shorten them, and does not rivet, but bends pins. Says side of backing touching tooth is already slightly countersunk by the pin of the punch; other side he countersinks. Solders as usual on sand and plaster. Thinks would warrant to solder 99 out of 160 without failure.

Ph.—His accidents are very rare; more frequent in vulcanite than in flat teeth. Countersinks backing on both sides; generally only bends pins. Likes alloy backing in preference to gold. Always solders on asbestos fiber.

W.—Rarely finds tooth breaking. Countersinks both sides; thinks fractures occur if backing does not perfectly fit back of tooth, for bad fit causes leverage. Does not rivet, and solders in sand and plaster. Thinks riveting a danger.

St.—Has very rarely pins break off or cracking; believes it is borax getting between tooth or backing, or overheating, which produces accidents. Always slightly rivets, and countersinks on side of rivet. Solders on plenty of sand and some plaster.

J.—Uses many thousands of teeth annually and does not have a tooth cracked or pins smashed once in three months. Thinks that he would use 99 out of every 100 teeth without any such mishap. Never rivets pins, nor cuts them short; only bends them over, and then file them down a little. Makes case red hot, after investing it in the usual way. Heats very gradually. Thinks fractures occur through overheating. Riveting tightly will be sure to make both go during soldering.

X.—Always countersinks holes both sides, and rivets before soldering. Has no accidents; if there are such, considers them due to tight riveting.

W.—Rarely or never has breakages of pins or cracking of teeth. When holes are punched in backing, he only slightly countersinks side nearest to tooth; the side of rivet he well countersinks, and then rivets slightly, as hard riveting causes a great strain and weakness. Cases when invested should be heated very gradually, and cooled very slowly. Non-attention to these points will bring about certain mishap. Thinks, as a rule, would fix 99 out of 100 teeth without failure. Has in hand the case of a lady who persists in having teeth fixed in and out; the same teeth have gone through the fire three or four times without the least accident.

H. M.—Does not have any teeth breaking off; if a breakage occurs, it is due to the bite; but accidents will arise if the backing is not brought close to the tooth.

Sometimes there is a bur at the holes, or a little porcelain bump is sticking up on the back of the tooth. Only bends pins; but if bite goes on pins, he rivets. Backing should be brought right up to cutting edge. Remove wax with boiling water, and of course heat gradually.

G. R.—Punches holes in backing; then countersinks on side where pins are bent; then cuts pins, so that when each is bent towards the other, they touch transversely. Cuts a little groove in backing with sculptor, files inside of each pin, and then they bend easily. Heats gradually; never has fractures of pins or cracking of teeth.

C. H.—Has had pins come off in flat and vulcanite teeth. Does not countersink on rivet side. Teeth sometimes give way months and years after making. He simply cuts pins off, and bends them together. Always solders on plaster alone.

H. A.—Rarely or never has breakage. Thinks men are careless in punching holes, and thus strain on pins breaks these off. If at any time he finds pins do not readily slip into the holes punched, at once uses fresh backing. Thinks some men in bending pins do it close to porcelain, and thus break the pins really before the case is soldered.

J. H, N.—Prepares backing by punching holes, which are countersunk on both sides and bent over, and sometimes slightly riveted. Pins and backing are carefully scraped. Fits tooth sometimes before and sometimes after backing. Accident the rarest thing. Considers failures due to riveting too hard and general carelessness.

R. T.—Takes backing, punching holes, and countersinks on side where he rivets. Always rivets, and does so on the side which goes on to the tooth; he then runs his sculptor across backing, taking in the two holes also. Sometimes he finds a little ridge of mineral near pins. In that case he bends backing a little to allow for it. Riveting ought to be done on lead, and the lead often changed, as it soon gets condensed. One ought to be careful not to use too much borax, as excess will cause accident if it gets behind backing. Never has teeth crack or the pins fracture.—*Journal British Assotiation.*

---

ARTICLE VIII.

### TREATMENT OF PROTRUSION OF THE LOWER TEETH.

